# Comorbid post-traumatic stress disorder in alcohol use disorder: relationships to demography, drinking and neuroimmune profile

**DOI:** 10.1186/s12888-017-1479-8

**Published:** 2017-08-29

**Authors:** Sudan Prasad Neupane, Jørgen G. Bramness, Lars Lien

**Affiliations:** 1 Norwegian National Advisory Unit on Concurrent Substance Abuse and Mental Health Disorders, Innlandet Hospital Trust, Post box 104, 2381 Brumunddal, Norway; 2 Norwegian Centre for Addiction Research (SERAF), University of Oslo, Oslo, Norway; 3 Department of Public Health, Hedmark University College, Elverum, Norway

**Keywords:** Alcohol use disorder, Post-traumatic stress disorder, Comorbidity, Cytokine, Tryptophan metabolism, Brain-derived neurotrophic factor

## Abstract

**Background:**

This study examined how alcohol use disorder (AUD) patients with post-traumatic stress disorder (PTSD) differed from those without PTSD in terms of demography, drinking patterns and C-reactive protein, inflammatory cytokines, tryptophan metabolism parameters, and brain-derived neurotrophic factor (BDNF).

**Methods:**

A consecutive sample (*N* = 187) of treatment-receiving AUD individuals were recruited from Nepalese facilities. They underwent fully structured psychiatric interviews. Serum levels of inflammatory cytokines [interleukin (IL)-6, IL-1 Receptor antagonist (IL-1Ra), IL-10, tumor necrosis factor-alpha (TNF-α), and interferon-gamma (IFN-γ)] were determined by a multiplex assay, kynurenine and tryptophan levels by high-performance liquid chromatography, and BDNF by enzyme-linked immunosorbent assay (ELISA).

**Results:**

The prevalence of exposure to severe trauma and PTSD was 74% and 17%, respectively. PTSD comorbidity was not associated with age, gender, or socioeconomic status, but with co-occurring major depression, history of attempted suicide, earlier peak of drinking problems, higher drinking quantity and withdrawal symptoms, experiencing alcoholic blackouts, and drinking problems among parents. None of the assessed neuroimmune parameters was related to comorbid PTSD.

**Conclusions:**

The findings support routine trauma screening in AUD treatment samples and screening for risky drinking in trauma populations to help guide interventions. The expected aberrations in neuroimmune functioning may not be found when examined in a sample with multiple psychiatric morbidities.

## Background

Exposure to extreme stress and subsequent development of post-traumatic stress disorder (PTSD) occurs frequently in persons with an alcohol use disorder (AUD), partly due to the associations between risky drinking and violence and accidents [1, 2]. Externalizing psychopathologies that are common in AUD, including attention-deficit/hyperactivity disorder, conduct, and antisocial personality disorders, can also promote impulsive and risk-taking behaviors leading to trauma [3], and increased anxiety and arousal among trauma-exposed AUD patients may increase susceptibility to the development of PTSD [4]. Conversely, repeated heavy drinking as a strategy to alleviate trauma symptoms may lead to AUD. A history of stressful life events increases the risk for AUD and increased AUD symptoms in affected individuals [5]. Prevalence studies show that up to half of all AUD treatment samples suffer from PTSD [6]. The prevalence of AUD in PTSD is also high. For example, in the US, 42% of PTSD subjects reportedly satisfied AUD diagnosis [7]. The odds ratio for PTSD in individuals with AUD reaches 4.9 in vulnerable groups such as ex-combatants [8]. The AUD-PTSD comorbidity is associated with greater severity of psychiatric symptoms and poorer treatment outcomes for either disorder [9–11]. Distinguishing the characteristics of patients with AUD who additionally have PTSD from those without comorbid PTSD thus has clinical implications.

Unlike most other psychiatric disorders, PTSD diagnosis requires the presence of a causative event (the trauma) and a specific symptom profile. Because of this etiological clarity, a rich body of research evidence has appraised the potential involvement of neuroendocrine, neurotransmitter, and neurotrophic systems in the pathophysiology of PTSD [12]. In spite of this progress, the biological mechanisms underlying AUD-PTSD comorbidity, such as the involvement of the immune system, await elucidation. Since early discovery of alcohol’s potent immune modulating properties [13], much research evidence has been gathered to identify the involvement of immune system in alcohol drinking behavior. There is now adequate evidence to suggest dysfunction of the innate immunity as an important underlying mechanism for addiction neurobiology both in animal models and in humans [14, 15]. For example, neuroinflammation and neurodegeneration subsequent to traumatic brain injury correlated with escalated alcohol self-administration in rats [16]. Postmortem brain specimens from alcohol-dependent humans have also shown altered expression of immune genes in multiple brain regions, including those that are involved in alcohol-drinking behavior [14]. Thus, the immune system has emerged as a crucial target for AUD treatment [17]. The effect of alcohol on the immune system depends on the ethanol dose [18], duration of drinking [19], and AUD severity [20, 21]. Chronic heavy drinking is associated with an increased risk of chronic conditions (e.g., cardiovascular diseases, liver cirrhosis) that are inflammatory in nature. Along the same lines, a review of PTSD research confirms elevated levels of inflammatory markers, including interleukin 1beta (Il-1ß), IL-6, tumor necrosis factor alpha (TNF-α), and interferon gamma (IFN-γ) in patients with PTSD compared to those in the healthy population [22, 23]. The former review also found Il-1ß and IL-6 levels to positively correlate with illness duration and PTSD severity, respectively. Further, one study reported that the pro-inflammatory cytokine IL-1ß levels were reduced following PTSD treatment, suggesting a biomarker value for L-1ß [24]. Also, recent prospective investigations have shown elevated levels of C-reactive protein (CRP), a commonly assayed acute phase protein, in survivors of childhood adverse events [25], and current CRP levels may predict the emergence of PTSD symptoms after trauma exposure later in life, implying a positive and prospective association between enhanced inflammation and the development of PTSD [26]. In a recent review, Olff and van Zuiden [27] suggested that post traumatic biological dysregulation, particularly alteration of the diurnal cortisol rhythm and IL-1 ß levels, may become more apparent over time. These lines of research implicate neuroimmune involvement in lifetime PTSD that is not necessarily limited to active symptoms of PTSD in affected individuals.

Whether the comorbidity between PTSD and AUD accompanies a neuroimmune profile that is predominantly proinflammatory in nature, and whether the added morbidity represents an aggravated proinflammatory state, remains unknown. Furthermore, it is unclear whether the correlates of comorbid PTSD in AUD are uniform across different countries and ethnicities. Tryptophan degradation along the kynurenine pathway by causing the release of neurotoxic metabolites is reported to be increased in stress-related psychiatric disorders [28]. Reduced neurogenesis and a lack of neurotrophic support, such as that reflected in reduced plasma brain-derived neurotrophic factor (BDNF) levels, as well as increased stress hormones are consistent findings in stress-related disorders, including PTSD [29, 30].

Activated innate immune response is also noted in other psychiatric disorders, such as major depression (MD) and bipolar affective disorder, which are often comorbid with PTSD [31]. Therefore, the interaction of co-occurring disorders is important to consider in otherwise heterogeneous psychiatric patient populations. Recently, Lindqvist et al. [32] reported that the inflammatory rise in PTSD among war veterans could not be explained by early life stress or depressive symptomatology, suggesting independent associations between immune activation and PTSD pathophysiology. Although international research on alcohol-induced immune changes, the immune mechanisms for alcohol drinking behavior, and immune changes in PTSD abounds, we are not aware of studies that specifically examine inflammation in the context of AUD-PTSD comorbidity. Moreover, there is a dearth of knowledge on the relationships between PTSD and other psychiatric conditions in non-Western settings.

Nepal endured a nationwide armed conflict between 1996 and 2006, which left at least 16,000 people dead, many displaced, missing, tortured, detained, or threatened [33]. In the 1990s, more than 100,000 Bhutanese citizens of Nepali origin took refuge in Nepal [33]. Other circumstances, such as sex trafficking, natural disasters (mainly flooding, landslide, and earth quakes), adverse childhood events, as well as socioeconomic inequality are potential contributors to the PTSD burden in Nepal. A few studies from Nepal have reported the prevalence of PTSD among vulnerable groups, such as tortured refugees (14%), former child soldiers (55%), and victims of political violence (14%) [34] and human trafficking (30%) [35]. In a sample of patients admitted for treatment and rehabilitation of drinking problems in eight different institutions in Nepal, we reported sociodemographic, drinking-related and neuroimmune correlates of comorbid depression [36–38]. We identified positive associations between inflammatory cytokines and lifetime MD, but not recent symptoms of depression, in the AUD sample [20]. In this study, we hypothesized that AUD patients exposed to potentially life threatening trauma, and those with PTSD comorbidity have an aggravated drinking problem as well as dysregulated neuroimmune function. Thus, we set out to investigate the prevalence of PTSD, and its socio-demographic and AUD-related correlates in a treatment sample of AUD in Nepal. Specifically, we examined the relationship between AUD-PTSD comorbidity and serum levels of CRP, inflammatory cytokines, tryptophan metabolism parameters, and BDNF.

## Methods

### Study setting and participants

This study was conducted in late 2010, long before the major earthquakes hit Nepal in April and May, 2015. Trauma psychiatry is only in its infancy, partly because of resource limitations and poor local constructs for PTSD hindering treatment seeking [39]. The nation’s specialized psychiatry and addiction treatment facilities are concentrated in the major cities and serve patients from across the country.

This study was carried out in eight institutions specialized for the treatment and rehabilitation of drug and alcohol-related problems in the Kathmandu and Lalitpur districts of central Nepal. Seven institutions were rehabilitation centers operating on non-pharmacological methods of care and one was a tertiary hospital. One of the rehabilitation centers exclusively served women, while the remaining centers, accepted only male patients. The hospital would receive patients with acute and chronic physical problems related to heavy drinking, whereas, the rehabilitation centers were often used by self-motivated users or their family to achieve abstinence using nonpharmacological methods. All treatment was based on payment made out of pocket. The rehabilitation centers were comparable in terms of user fees, and treatment modality. However, the hospital-based patients were likely to have different physical health profiles than patients recruited from the rehabilitation centers. The participant recruitment procedure and the participant characteristics from the original study have been published previously [38, 40]. In short, persons receiving residential treatment at the centers between August and December, 2010, were invited to participate in the study. Among these participants, 95% provided consent. The first author collected blood samples at least 4 days (mean 34.4, SD 32.7) after the last alcohol intake and conducted fully structured psychiatric interviews after 10 days in the treatment programs. Patients were undergoing treatment for a mean of 54.9 days (SD 47.2), and a great majority of them (86%) were from a rehabilitation center setup.

Data were available on 187 participants. Only 20 (11%) participants, including 10 Bhutanese refugees, were women. The mean age of the participants was 35.5 years (SD 10.1, range 14–63). At an average of 30.0 years of age (SD 10.2), female participants were significantly younger than their male counterparts at 36.2 years of age (SD 9.9). Participants provided written informed consent before enrolment. Those unable to read or write (eight men and eight women) were read out the contents of the information sheet (Nepali language) individually by the first author. Then, the potential participant was given a chance to ask any further questions pertaining to the study and their participation. Those willing to participate were asked to provide a thumbprint with a witness (treatment staff or patient party)'s signature, confirming that any of the participant’s queries had been answered by the researcher and that the consent was given freely. The study was approved by the Regional Committee for Medical Research Ethics of Norway and the National Health Research Council of Nepal.

### Measures

We used the Composite International Diagnostic Interview (CIDI)-version 2.1 [41], a fully structured psychiatric interview questionnaire, available in the Nepalese language to capture the demographic details, and to generate the Diagnostic and Statistical Manual for Mental Disorders, Fourth Edition [42] compatible diagnoses of alcohol abuse or alcohol dependence (together referred to as AUD in this paper), major depressive episodes, and PTSD. Patients satisfying either alcohol abuse and/or alcohol dependence criteria are together defined as having an alcohol use disorder. For elucidation of drinking pattern, we also used the Alcohol Use Disorder Identification Test (AUDIT) [43]. AUDIT is a 10-item questionnaire developed by the World Health Organization to easily screen for excessive drinking and to assist in brief interventions for alcohol-related problems [44]. This instrument has demonstrated reliability and validity in a similar setting to this study [45]. The conversion table available in the Nepali version of the CIDI questionnaire was used to calculate standard units of drinks in units of ethanol. Thus, a bar-served glass of *Raksi* (distilled local drink) was considered 2 units of ethanol and 1 *mana* (approximately 0.55 L) of *Jand* (domestically fermented beverage) was calculated as containing 3 ethanol units. The abstinence duration was determined by inquiring the most recent alcohol consumption episode, and participants responded to whether or not they had ever engaged in driving under the influence of alcohol.

For assessment of trauma symptoms, the CIDI has 10 categories of adverse life events and an unclassified extremely stressful event with responses in yes/no options to indicate the worst traumatic event along with PTSD symptoms and time specifics. Symptoms consequent to trauma exposures were assessed using the time qualifier ‘ever’ to identify lifetime PTSD. PTSD resulting from two or more episodes of any of the trauma types: armed combat, sexual assault/rape, and threatened with weapon/kidnapped/held captive or tortured was defined as complex trauma PTSD [46]. Other forms of trauma were defined as type I trauma. Additionally, depression and suicidality modules of CIDI were utilized to operationalize related variables. Suicidal ideation captured the individual’s serious thoughts about taking their own life and suicidal attempt was defined as unsuccessful attempts of taking one’s own life. Current distress symptoms were assessed by the Hopkins Symptoms Checklist-25 [47], an instrument that has 10 items in the anxiety domain and 15 items in the depression domain, with each item to be scored from none (1) to extremely (4). Current distress was defined as an average score above the conventional cutoff of 1.75 in the Checklist [48]. Family history of drinking problems and current smoking was asked in a separate pro forma, and history of other substance misuse was captured by section L (Illegal substance) of the CIDI.

### Blood sampling and laboratory assays

Venous blood samples were collected in preservative-free BD Vacutainer® gel tubes on site. Within 2.5 h of collection, the blood sample was centrifuged on a swing-out centrifuge device at 1300×*g* at room temperature. The separated serum was transferred into a polypropylene tube and immediately frozen in a refrigerator under −70 °C. The samples were packaged to UN packaging standards and transferred to Norway by air courier using dry ice and stored frozen until assay.

Serum concentrations of IL-1 receptor antagonist (IL-1ra), IL-6, IL-10, TNF-α, and IFN-γ were determined as part of a Bio-Plex protein array system (Human Bio-Plex; Bio-Rad Laboratories Inc., Hercules, CA, USA) based on the xMAP multiplex technology (Luminex, Austin, TX, USA). Serum tryptophan and kynurenine levels were determined by high-performance liquid chromatography, using an ultraviolet absorption detector for kynurenine and a fluorescence detector for tryptophan on Agilent Infinity 1290 systems (Agilent Technologies, CA, USA). The ratio of kynurenine to tryptophan concentrations × 10^3^ (KT ratio) was calculated and used as a measure of the tryptophan degradation index. Serum BDNF concentration was determined by enzyme-linked immunosorbent assay (ELISA), using a commercially available kit Human BDNF Quantikine ELISA kit (R&D Systems, Minneapolis, MN, USA) based on a sandwich enzyme immunoassay technique. Steps for analysis were followed according to the manufacturer’s instructions. All analyses were performed by technicians blinded to the clinical data.

### Analysis plan

Descriptive data are presented as counts (%), mean (SD), and median (interquartile range). The statistical differences between the no PTSD and PTSD groups were, as was the case for other categorical variables, calculated by Pearson’s chi-squared test. Student’s *t*-test or Mann-Whitney *U*-test was used for continuous variables according to normality of data distribution. Possible differences in the levels of neuroimmune parameters between patients with and without PTSD (the main outcome measure) were estimated by the Mann-Whitney *U*-test. Further, the Kruskal-Wallis test was used for examining the differences in the neuroimmune parameters across the three groups of patients: trauma naïve, traumatized non-PTSD, and lifetime PTSD patients. The significance level was set at *P* = 0.05. The statistical analysis was performed using IBM SPSS Statistics for Windows version 22 (Armonk, NY, USA).

## Results

Thirty-two participants (17%) satisfied DSM-IV criteria for lifetime PTSD.

### Traumatic experience and post-traumatic symptoms

A positive history of traumatic events was reported by 139 participants (74%). Serious road traffic accidents constituted the most frequent trauma type and a substantial proportion of PTSD cases were attributed to this trauma type (Table 1). Fifty-six per cent of the participants reported a positive history of driving under the influence of alcohol. Events that most frequently resulted in PTSD were torture (53%), being threatened with a weapon/kidnapped/held captive (39%), and sexual assault (37%). Eleven patients (6%) satisfied the defined criteria for complex trauma PTSD.

**Table 1:** Traumatic experience and prevalence of PTSD among Nepalese patients with alcohol use disorder

Type of traumatic experience	Prevalence of exposure (*N* = 187) n (%)	Prevalence of PTSD among exposed n (%)	Worst trauma^a^ n	Exposure frequency mean (range)	Age at first exposure mean (SD) years
Military combat	7 (3.7)	0 (0)	2	3.5 (3–4)	25.0 (4.2)
Serious road traffic accident	87 (46.5)	21 (24.1)	43	1.3 (1–6)	24.9 (8.5)
Natural disaster	25 (13.3)	5 (20.0)	9	1	14.8 (5.6)
Witnessed killing/serious injury/violence	45 (24.1)	12 (26.7)	18	1.3 (1–4)	23.2 (6.5)
Rape	6 (3.2)	2 (33.3)	2	1	20.5 (4.9)
Sexual assault other than rape	8 (4.3)	3 (37.5)	1	2	28.3 (7.4)
Assaultative violence	37 (19.8)	12 (32.4)	17	1.9 (1–11)	28.1 (6.1)
Threatened with weapon/kidnapped/held captive	33 (17.6)	13 (39.4)	9	1.8 (1–5)	24.8 (6.7)
Tortured	17 (9.1)	9 (52.9)	11	1.7 (1–5)	22.2 (7.5)
Other serious trauma	56 (29.9)	17 (30.4)	21	1.4 (1–5)	23.6 (7.3)
Sudden, unexpected death of relative/friend	25 (13.3)	8 (32.0)	25	1.5 (1–4)	25.7 (7.2)

^a^figures in the column do not add up to total subjects due to missing data (*n* = 29)

Among trauma-exposed participants, re-experiencing symptoms were present in 72 participants (52%), hyper-arousal symptoms were present in 51 participants (37%) and avoidance/numbing symptoms were present in 47 participants (34%). Forty-one participants (30%) had experienced symptoms of all three clusters.

### PTSD and associated factors

Patients with lifetime PTSD more often had an earlier peak of drinking problems, had higher average daily drinks, experienced alcoholic blackouts, reported higher alcohol-related psychological illness, and reported drinking problems among parents (Table 2). Patients with PTSD had a greater variety of trauma (2.9 SD 1.7) than those without PTSD (1.5 SD 1.4) (*p* < 0.001). They also had a higher exposure frequency of traumatic events (2.1 SD 2.0 vs. 1.3 SD 0.8; *p* = 0.007). Age at traumatic experience was unrelated to later PTSD. The only PTSD symptom cluster associated with the level of AUDIT score was avoidance/numbing [t(179) = 2.22, *p* = 0.028].

**Table 2:** Associations of sociodemographic and clinical characteristics with post-traumatic stress disorder among Nepalese patients with alcohol use disorder

		Total	No-PTSD	PTSD		
Variables		*N* = 187	*N* = 155	*N* = 32	Statistical value (df)	*P-value*
Age (years)	mean (SD)	35.5 (10.1)	35.9 (10.4)	33.1 (7.9)	1.40 (178)	0.163
Male sex	n (%)	167 (89.3)	139 (89.7)	28 (87.5)	0.13 (1)	0.717
Urban living	n (%)	101 (56.1)	81 (53.6)	20 (69.0)	2.31 (1)	0.128
Unmarried	n (%)	70 (38.9)	59 (39.1)	11 (37.9)	0.01 (1)	0.908
Higher caste	n (%)	104 (57.8)	92 (60.9)	12 (41.4)	3.81 (1)	0.051
Higher education	n (%)	128 (71.1)	111 (71.6)	22 (68.89	0.11 (1)	0.745
High income	n (%)	104 (66.7)	84 (65.1)	20 (74.1)	0.81 (1)	0.369
Adequate social support	n (%)	129 (68.9)	110 (72.8)	19 (65.5)	0.64 (1)	0.422
Age at onset of habitual drinking (years)	mean (SD)	17.9 (5.7)	17.8 (5.6)	18.3 (6.3)	−0.43 (185)	0.672
Age at peak drinking problem	mean (SD)	34.0 (9.9)	34.6 (10.0)	30.8 (8.7)	2.02 (185)	**0.045**
Drinking career (years)	mean (SD)	19.9 (9.9)	17.6 (9.9)	14.4 (9.6)	1.70 (179)	0.091
Daily drinking units	mean (SD)	17.6 (14.5)	16.4 (12.4)	22.8 (20.5)	−2.29 (167)	**0.024**
Number of alcohol withdrawal symptoms	mean (SD)	5.9 (3.7)	5.7 (3.8)	7.2 (3.4)	−2.22 (170)	**0.032**
Drinking >2 days/week	n (%)	162 (86.6)	136 (87.7)	26 (81.3)	0.97 (1)	0.390
Drinking units/typical day ≥5	n (%)	132 (72.1)	110 (71.0)	22 (68.8)	0.29 (1)	0.802
Guilt/remorse on drinking	n (%)	134 (71.7)	110 (71.0)	24 (75.0)	0.21 (1)	0.645
Eye-opening	n (%)	137 (76.5)	113 (75.3)	24 (82.8)	0.75 (1)	0.388
Alcohol-induced blackouts	n (%)	110 (62.1)	87 (58.8)	23 (79.3)	4.34 (1)	**0.037**
Alcohol use related police arrests	n (%)	63 (35.0)	51 (33.8)	12 (41.4)	0.62 (1)	0.432
Used alcohol more often than intended	n (%)	148 (82.2)	123 (81.5)	25 (86.2)	0.38 (1)	0.540
Alcohol related physical illnesses	n (%)	119 (65.4)	99 (65.6)	20 (64.5)	0.01 (1)	0.911
Alcohol related psychological illnesses	n (%)	132 (70.6)	16 (50.0)	116 (76.8)	9.45 (1)	**0.002**
Parental problem drinking	n (%)	68 (37.8)	52 (34.49)	16 (55.2)	4.45 (1)	**0.035**
Currently smoking (yes)	n (%)	121 (65.1)	100 (64.9)	21 (65.6)	0.01 (1)	0.941
Other substance misuse (ever)	n (%)	58 (31.0)	49 (31.6)	9 (28.1)	0.15 (1)	0.698
Prior treatment relapse	n (%)	40 (21.4)	33 (21.3)	7 (21.9)	0.01 (1)	0.941
Currently distressed	n (%)	49 (27.8)	41 (27.9)	8 (27.6)	0.01 (1)	0.973
Major depression (life time)	n (%)	85 (45.4)	63 (40.9)	22 (68.8)	8.28 (1)	**0.004**

Test statistics were calculated by Chi-squared test, Student’s *t*-test or Mann Whitney *U*–test; significant *P*-values are given in bold

PTSD in the sample was related to several other clinical features. Association between lifetime MD and lifetime PTSD was significant (χ^2^ = 8.28, df = 1, *p* = 0.004). Examining the associations between MD and PTSD revealed that only with hyperarousal cluster of PTSD symptoms was MD in the sample related (χ^2^ = 4.35, df = 1, *p* = 0.037). Furthermore, PTSD patients were more likely to report a positive history of suicidal ideation and attempted suicide (χ^2^ = 4.98, df = 1, *p* = 0.014). Although patients with PTSD scored higher on AUDIT (30.4 ± 8.9 vs. 27.4 ± 8.5), the difference was not statistically significant [t(179) = −1.70; *p* = 0.096]. PTSD was more common among patients attending a hospital psychiatric unit than among patients attending rehabilitation centers (OR = 3.8 (95% CI 1.5–9.7), *p* = 0.004). At the end of the interview, we asked patients if they had experienced additional trauma to those in the questionnaire (see Table 1), for which 55 individuals (29%) responded positively. Notably, only this unclassified trauma exposure was significantly related to comorbid MD (χ^2^ = 14.34, df = 1, *p* < 0.001).

### Neuroimmune parameters in trauma exposure and PTSD

The cytokines, CRP, tryptophan degradation index and BDNF levels in trauma naïve, trauma exposed, and PTSD groups did not vary significantly (Table 3). The levels of these neuroimmune factors were not different between patients with and without PTSD. Neither did a difference exist in neuroimmune factor levels between patients with type I trauma and trauma associated with complex trauma PTSD. These parameters were not specifically related to re-experiencing symptoms, hyper-arousal symptoms, avoidance/numbing PTSD symptom clusters, to any specific or nonspecific traumatic exposures, the number of different trauma exposures, or abstinence duration/treatment duration (data not shown in table).

**Table 3:** Neuroimmune parameters in alcohol use disorder patients by trauma exposure and PTSD status

	No traumatic history (*n* = 47)	Trauma without PTSD (*n* = 107)	PTSD (*n* = 32)	Statistical value (df)	*P-value*
Parameter	Median (IQR)	Median (IQR)	Median (IQR)		
CRP	0.5 (0.5–4.0)	1.0 (0.5–4.0)	1.0 (0.5–2.0)	0.41 (2)	0.813
IL-1RA	112 (73.6–142.7)	113 (88.6–147.0)	110 (88.6–134.1)	0.74 (2)	0.691
IL-6	8.6 (6.8–10.9)	9.5 (7.4–11.3)	9.1 (7.3–10.9)	0.39 (2)	0.825
IL-10	9.5 (6.5–14.2)	9.7 (7.2–14.0)	10.2 (6.5–14.5)	0.38 (2)	0.828
TNF-a	32.6 (25.0–38.8)	31.9 (25.0–44.1)	33.8 (25.0–40.4)	0.196 (2)	0.906
IFN-g	90.1 (71.2–118.4)	109.7 (77.3–136.5)	104.9 (70.7–134.2)	2.938 (2)	0.230
Kynurenine/tryptophan ratio	41.8 (33.7–51.9)	44.5 (35.2–53.6)	42.3 (31.4–47.9)	2.213 (2)	0.331
BDNF	22.6 (17.3–31.8)	25.5 (18.8–31.4)	24.3 (21.2–31.3)	0.343 (2)	0.842

Reported test statistics were calculated by Kruskal–Wallis Test

Because of the underrepresented female sample, we performed post hoc analysis to confirm the consistency of the observed findings. Excluding females from the data did not alter any significant findings.

## Discussion

In this study of inpatients receiving treatment for drinking problems in Nepal, the prevalence of PTSD comorbidity was 17%. While demographic and neuroimmune differences were not found between AUD patients with and without lifetime PTSD, features indicating greater overall drinking problems were associated with comorbid PTSD.

The rates of comorbidity are similar to those reported in a recent study among inpatients with alcohol dependence in a suburban setting in the USA (19%) [49], multiple addiction treatment centers in Germany (15%) [10] and Poland (25%) [50], an inpatient addiction treatment unit in the Netherlands (26%) [51], substance abuse in- and outpatients in Norway (16%) [52] and well within the range of 15–30% reported by several other studies [6]. Nearly three quarters of patients had traumatic experiences, a proportion similar to that reported by other investigators [50, 51]. Some inconsistency in the prevalence figures reported in literature may owe partly to researchers choosing to focus on lifetime diagnosis or on recent symptoms of PTSD. Traumatized women are more likely to develop PTSD compared to men [53], but our sample did not reveal this, likely owing to underrepresented female participants. Among the symptom clusters of PTSD, our data found evidence for associations between avoidance/numbing and heavy drinking, a finding supported by the literature [6]. Trauma-exposed individuals are thought to have a higher risk for PTSD in case of severe trauma, lack of social support, additional life stress, low socioeconomic status and psychiatric history in the family and self, but the contribution of these pre-and post-traumatic vulnerability factors for PTSD development is unclear because of a wide heterogeneity in the studied samples [53]. Thus, investigations of clinical and neuroimmune correlates of PTSD must also consider biopsychosocial harms associated with prolonged repeated trauma different from isolated type I trauma [46]. One may anticipate factors related to drinking as potential explanations of PTSD in AUD individuals. Indeed, the comorbid group patients had more alcohol problems as depicted by consistently greater likelihood of high frequency and intensity drinking, and associated symptoms of alcohol dependence as well as impairments in several life domains, including greater suicidality. It should, however, be noted that the response regarding suicidality might only reflect events related to the most severe episode of depression and the data may have suffered reporting and/or measurement bias concerning suicidal attempts that in reality may range from manipulative gestures, to cries for help to unsuccessful lethal acts. One way of explaining higher alcohol use among PTSD subjects is through self-medication strategy [6, 54]. However, to what extent our data confers to this explanation is unclear. The vicious cycle of risky drinking leading to increased vulnerability for trauma that again reinforces higher alcohol seeking attitudes to relieve trauma symptoms must be considered. A longitudinal study design is needed to confirm a causal pathway while also accounting for other externalizing psychopathology.

Although the intoxication debut age was similar in both groups, PTSD subjects had an earlier peak of drinking problems. Additionally, serious road traffic accidents were the most predominant traumatic event and comprised the trauma category containing the most cases of PTSD in the sample. Given the high rates of self-reported driving under the influence of alcohol in the sample (56%), it is likely that road traffic accidents as a consequence of risky drinking comprised the traumatic event leading to PTSD in the cases of many of the participants. However, the design of the study does not allow any causal inferences, and this assertion should be considered speculative, but comprises an area that is an important consideration for future studies because this bears a potential for intervention strategy at the public health level. Although systematic investigation on driving under the influence of alcohol is pending [55], the high rates of road traffic accidents in the AUD sample suggests a need to increase focus on continued implementation of alcohol testing on vehicle drivers as a viable strategy to control road traffic accidents. The study was carried out in the aftermath of armed conflict between the government forces and the Maoist-rebels and showed clear wounds of the conflict: torture and being threatened with a weapon, kidnapped, or held captive as the main events associated with PTSD.

In the studied population, we have earlier shown lifetime MD to be related to higher inflammatory cytokine levels [20], whereas, recent depressive symptoms were related to lower BDNF levels [36] as well as altered tryptophan metabolism parameters [37] in the serum. Despite being highly associated with MD history, lifetime PTSD was not related to any of the assessed cytokines, CRP, or other neuroimmune factors. This finding has several implications. Firstly, as observed in general psychiatric populations [56], post-traumatic and depressive disorders cluster in AUD individuals. Secondly, although depression and PTSD diagnoses are commonly comorbid and may seem to reflect parts of a common generic archetype of post-stress syndrome in the susceptible AUD patients [57], neuroendocrine responses [58] and, possibly, neuroinflammatory processes in depression and PTSD could differ widely. The findings of robust associations between PTSD diagnosis and an inflammatory state regardless of depression comorbidity in a previous study [32] may possibly be a reflection of the pro-inflammatory processes in PTSD specific to complex trauma, thus not applicable to our sample. Thirdly, an absence of raised inflammatory markers in PTSD in our sample may simply imply heterogeneity and insufficient size of the comorbid group, resulting in type II error [59]. A fourth possibility is that, an interaction effect between different dimensions of PTSD and depression may nullify the proinflammatory activity otherwise widely reported in isolated disorders [22]. Finally, the distinct changes reported in the literature are often based on samples exclusive of comorbid substance use disorders and thus may not represent accurate observations in many psychiatric populations with multiple disorders. We have earlier reported partly unexpected findings on tryptophan metabolism parameters in the same study population, whereby tryptophan levels were higher and tryptophan degradation was lower in co-morbidly depressed individuals compared to the non-depressed AUD sample [37], and this contradictory finding is not unique. It has been widely discussed that inflammation may be neither necessary nor sufficient for depression to occur when referring to a wide heterogeneity of clinical populations [60]. We performed post hoc analysis to examine whether any of the symptom clusters of PTSD or the complexity of trauma could explain the absence of associations between PTSD and inflammatory markers. Neither subgroup analysis on symptom clusters nor the complexity of trauma changed the results, and there was no association between the observed neuroimmune factors and the number of different traumatic exposures. There could also be other explanations for the negative finding, such as the focus on lifetime PTSD diagnosis instead of ongoing symptoms. In fact, comorbid MD was related only with the hyperarousal cluster of PTSD symptoms, probably due to increased overlap of physiological hyperarousal symptoms (e.g., altered sleep and difficulty concentrating) that are non-specific to the insulting trauma between MD and PTSD patients. Association between unclassified trauma exposure and comorbid MD in the sample could reflect the patient’s attribution of the most severe psychological stressor.

Chronic heavy drinking modulates the innate immune system, and given that the sample had a varying degree of AUD severity and some of the subjects possibly had physical organ damage associated with AUD, which may have resulted in altered immune response [21]. Thus, a distinctive neuroimmune profile may not be observed uniformly among either group. However, it should be noted that the findings are not unanimous in regard to the inflammatory profile of PTSD subjects. For example, in Iraqi refugees in Sweden, circulating CRP levels were found to be lower among PTSD subjects compared with controls [61]. Thus, a proinflammatory or a neurodegenerative profile may be absent in PTSD due to other confounders, such as ongoing infection, medication, body fat content, organ damage, physical activity, and ethnic differences [62, 63]. In a recent meta-analytic review of cytokines in PTSD, Passos et al. noted that the discrepancy between findings across studies on inflammatory markers in PTSD could be explained through the modulatory effects of psychotropic medication and the occurrence of comorbid major depressive disorder [22]. Yet another mechanism which led to comparable inflammatory cytokine levels between patients with and without PTSD could be the extent of alcohol misuse. High doses of alcohol causes an increase in gut permeability, which causes bacterial lipopolysaccharide translocation in the body, subsequently causing cytokine upregulation [64]. This could have been attenuated by post-traumatic tolerance to lipopolysaccharides [65].

We are not aware of other studies that have specifically investigated neuroimmune factors in PTSD in the context of AUD, which precluded any comparisons to the literature. As in all studies, caveats must be borne in mind. This cross-sectional study cannot imply a causal association between inflammation, trauma, and other clinical measures. The study sample was not selected for PTSD, and thus, a limited number of participants were available for subgroup analysis. Despite being allocated to distinct groups, women, hospital inpatients, and participants with refugee backgrounds (all women) were underrepresented in the sample. This limited the generalizability of the study findings. All participants had an AUD which is, in itself, a modulatory factor for neuroimmune status. We are unable to confirm the accuracy of recall of past adverse events and other forms of reporting bias as several of the variables were constructed from personally sensitive self-report data. Further, calculation of standard alcohol units is approximate as the possibility of a wide variation in the ethanol concentration of locally brewed beverages cannot be ruled out. Future adequately sampled studies should account for confounders of inflammatory mediators in blood, and the comparison group should include a healthy control as well as isolated disorders. Epigenetic changes relevant to hypothalamic pituitary adrenal axis response have been found to correlate with specific childhood abuse and its repetitiveness [66]. Specific trauma types, trauma complexity, number of adverse life events, trauma severity, and duration as well as recency of PTSD symptoms are important considerations for future studies of trauma psychoneuroimmunology.

## Conclusions

Our data from a contextually different setting than most other studies in the field provides preliminary insights to extend our understanding of the complex comorbidity between AUD and PTSD, and contributes to the accumulating data on neuroimmune alterations in such comorbidities. PTSD comorbidity in patients with AUD in Nepal was associated with co-occurring major depression, history of attempted suicide, earlier peak of drinking problems, higher drinking quantity and withdrawal symptoms, experiencing alcoholic blackouts, and drinking problems among parents. These findings support the need for routine trauma screening in AUD treatment samples, screening for risky drinking in trauma populations, and planning interventions accordingly. Additionally, the findings show that psychoneuroimmunological investigations do not necessarily reveal expected aberrations in neuroimmune functioning when examined in a sample with multiple morbidities.

## Abbreviations

AUD: Alcohol use disorder
BDNF: Brain-derived neurotrophic factor
CIDI: Composite International Diagnostic Interview
CRP: C- Reactive Protein
IFN-γ: Interferon gamma
Il-1ß: Interleukin 1beta
IL-6: Interleukin-6
MD: Major depression
PTSD: Post-traumatic stress disorder
TNF-α: Tumor necrosis factor alpha
